# A53 NOVEL PROTEASE–HISTAMINE INTERACTIONS POTENTIATE PAIN SIGNALING EVOKED BY FECAL SUPERNATANTS FROM IBS PATIENTS

**DOI:** 10.1093/jcag/gwae059.053

**Published:** 2025-02-10

**Authors:** N Jimenez Vargas, D A Gilmour, B Sokrat, A S Bennett, A E Lomax, D E Reed, N W Bunnett, S J Vanner

**Affiliations:** Queen’s University, Kingston, ON, Canada; Queen’s University, Kingston, ON, Canada; New York University, New York, NY; Queen’s University, Kingston, ON, Canada; Queen’s University, Kingston, ON, Canada; Queen’s University, Kingston, ON, Canada; New York University, New York, NY; Queen’s University, Kingston, ON, Canada

## Abstract

**Background:**

Proteases and histamine, originating from intestinal tissue and/or the microbiota, have each been implicated in the severe pain symptoms reported by patients with irritable bowel syndrome (IBS). However, the effects of their combined signaling and the mechanisms involved have not been studied.

**Aims:**

To determine whether histamine and trypsin synergistically enhance colonic nociceptive signaling and, if so, to study the mechanisms involved.

**Methods:**

Fecal supernatants (FS) were obtained from IBS patients with low and high pain scores. Responses to luminal application of FS, histamine and protease (trypsin) and antagonists (H1R-pyrilamine, PAR2-GB83) were studied using *ex vivo* mouse colonic afferent nerve recordings. In mechanistic studies, excitability of isolated mouse dorsal root ganglia (DRG) neurons was measured using patch clamp recordings of rheobase after combined or sequential application of agonists. To study endosomal signaling, neurons were incubated with clathrin inhibitor, pitstop2, before agonists. Using HEK cells, recruitment of mGαq to PAR2 and H1R at the plasma membrane (CAAX) or early endosome (Rab5) was measured using bioluminescence resonance energy transfer (BRET) in response to sequential application of increasing doses of trypsin and histamine.

**Results:**

In colonic afferent nerve recordings, FS from IBS patients with high pain scores increased mechanosensitivity (50%; *p* <0.001) and this was blocked by either a PAR2 and H1R antagonist, whereas FS from those with low pain scores had no effect. Trypsin and histamine at subthreshold concentrations (30 μM) alone had no effect. However, their co-application increased afferent mechanosensitivity (46%; *p* <0.001); similar effects were observed in patch clamp recordings from DRG neurons (rheobase decreased 33%; *p* <0.05). To study the mechanism of this synergistic interaction, DRG neurons were recorded after a sequential incubation with histamine and proteases. Incubation first with histamine had no effect (*p*=0.55) on the subsequent trypsin-mediated PAR2 excitability but incubation first with trypsin amplified the histamine response by 42 % (p<0.01). This PAR2-mediated action was blocked by inhibitors of endosomal signaling. In keeping with these findings, BRET studies revealed that incubation first with trypsin promoted mGαq recruitment to the plasma membrane and early endosomes, whereas histamine did not.

**Conclusions:**

Conclusion: FS from IBS patients with high pain scores caused exaggerated pain signaling from the mouse colon and was mediated by the synergistic action of histamine and proteases. Mechanistic studies revealed a novel pathway where trypsin-mediated PAR2 endosomal signaling amplified histamine-evoked pain signaling. These findings could inform future studies of IBS therapies and biomarkers.

**Funding Agencies:**

CIHR

